# The CEBPE rs2239633 genetic polymorphism on susceptibility to childhood acute lymphoblastic leukemia: an updated meta-analysis

**DOI:** 10.1186/s12199-020-00920-2

**Published:** 2021-01-04

**Authors:** Jin Liu, Gu Weiling, Li Xueqin, Xie Liang, Wang Linhong, Chen Zhongwen

**Affiliations:** 1 Department of Control and Prevention of Chronic Non-communicable Diseases, Jiaxing Center for Disease Control and Prevention, Jiaxing, Zhejiang, 314050 China; 2 Office, Jiaxing Center for Disease Control and Prevention, No.486 Wenqiao Road, Jiaxing, Zhejiang, 314050 China

**Keywords:** *CEBPE* rs2239633, Polymorphism, Childhood acute lymphoblastic leukemia, Meta-analysis

## Abstract

**Objectives:**

We performed an updated meta-analysis to clarify the relationship between the CEBPE rs2239633 polymorphism and the childhood acute lymphoblastic leukemia (CALL) susceptibility.

**Methods:**

All the case-control studies were updated on October 5, 2020, through Web of Science, PubMed, Cochrane Library, Embase, and China National Knowledge Infrastructure (CNKI) electronic database. The heterogeneity in the study was tested by the *Q* test and *I*^2^, and then the random ratio or fixed effect was utilized to merge the odds ratios (OR) and 95% confidence interval (CI). We also performed sensitivity analysis to estimate the impact of individual studies on aggregate estimates. Publication bias was investigated by using funnel plot and Egger’s regression test. All statistical analyses were performed using Stata 12.0.

**Results:**

A total of 20 case-control studies were selected, including 7014 patients and 16,428 controls. There was no association of *CEBPE* rs2239633 polymorphism with CALL (CC vs CT + TT: OR = 1.08, 95% CI = 0.94–1.26; CC + CT vs TT: OR = 1.10, 95% CI = 0.94–1.30; C vs T: OR = 1.02, 95% CI = 0.92–1.13). In the subgroup analysis by ethnicity, there is no significant association of this polymorphism and CALL risks among Asian and Caucasian populations in the three genetic models (CC vs CT + TT, CC + CT vs TT, and C vs T).

**Conclusion:**

This meta-analysis found no significant association between the CEBPE rs2239633 polymorphism and susceptibility to CALL.

## Introduction

Acute lymphoblastic leukemia (ALL) is a malignant disease of the blood system. It occurs mostly in children under 15 years of age. The peak age of onset was 2–5 years old [1, 2], accounting for about 1/3 of childhood malignant tumors [3]. Although the etiology and pathogenesis were not yet clear, previous studies had shown that ALL was the result of multiple factors such as genetic variation and exposure to carcinogens in the environment [4, 5]. In recent years, genome-wide association studies (GWAS) had shown that gene mononucleotide polymorphism (SNP) variation was an important risk factor for CALL [6–9].

The CEBPE gene was located on the human chromosome 14q11.2, which was a member of the CCAAT-enhancer-binding protein family, and its encoded protein belongs to the basic leucine transcription factor. The CEBPE gene-encoded protein was essential for terminal differentiation and functional maturation of myeloid committed progenitor cells, especially for the maturation of neutrophils and giant wah cells [10]. Mutations in CEBPE would cause loss of neutrophil granules [11]. Akasaka had reported that CEBPE mutations can cause translocation of immunoglobulin heavy chain chromosomes, which often occurred in children with B-precursor E cell leukemia [12]. This indicated that the CEBPE gene played an important role in the occurrence and development of ALL.

Two meta-analysis studies in 2014 [13] and 2015 [14] found the association of *CEBPE* rs2239633 polymorphism with the risk of CALL, but the conclusions obtained from the two studies were reversed. In addition, since 2015, many studies had reported *CEBPE* rs2239633 polymorphisms and the risk of CALL [11, 15–19]. Therefore, the purpose of this meta-analysis was to investigate the relationship between *CEBPE* rs2239633 polymorphism and the risk of CALL.

## Materials and methods

### Search strategies

We conducted a systematic online search of the literature in the Web of Science, PubMed, Cochrane Library, Embase, and China National Knowledge Infrastructure (CNKI) electronic database, covering relevant studies published until October 5, 2020. The keywords for the search were as follows: (“rs2239633” OR “*CEBPE*”) AND (“polymorphism” OR “variant” OR “mutation”) AND (“acute lymphoblastic leukemia” or “ALL”). The literature on relevant data was searched in English and Chinese, respectively. In addition, the retrieved articles and references were performed with manual searches. Referring to the Preferred Reporting Project (PRISMA) Guide for Systematic Evaluation and Meta-Analysis [20], an information flow diagram related to the final eligibility data was constructed by screening all retrieved literature.

### Inclusion and exclusion criteria

Screening for the studies of the relationship between *CEBPE* rs2239633 polymorphism and the risk of ALL is according to the following inclusion criteria: (1) the design of the study was case-control, (2) the full text can be found, (3) the genotype information of the *CEBPE* rs2239633 polymorphism was available, and (4) the relationship of the *CEBPE* rs2239633 polymorphism and the risk of ALL was evaluated. The major exclusion criteria were (1) not a case-control study; (2) repeating early publications (studies used in different publications for the same sample data, including only the most complete samples after careful review); (3) unpublished articles, conference papers, meta-analysis, and systematic reviews; and (4) family-based pedigree research. This meta-analysis strictly followed the requirements of the preferred reporting project for the systematic review and meta-analysis guidelines [20].

### Data extraction

The analysis data of the selected studies were independently extracted by two researchers using standard data collection forms. Study-related information extracted from each literature was as follows: first author, year of publication, country of origin, mean age and gender in cases and controls, numbers of cases and controls, Hardy-Weinberg equilibrium, genotyping method, source of controls, and available genotype frequency information for *CEBPE* rs2239633. If the same sample data appeared in multiple publications, only the publication with the largest sample size was included in the study. The differences between the two investigators were resolved through discussion. If the discussion could not resolve the objection between the two, the objection would be judged by the third investigator. All data were obtained from the full text of the published research, and the author was not contacted for further information.

### Study quality assessment

Two evaluators evaluated the quality of the included studies according to the Newcastle-Ottawa Scale (NOS) [21], which was applicable to the quality assessment of observational studies. The difference between the two evaluators was reported and resolved by the third evaluator. The scores of research quality mainly included the following three aspects: (1) selection of the case groups and control groups (4 stars), (2) quality of confounding factor correction in case and control population (2 stars), and (3) determination of the exposure of interest in the studies (3 stars). For each item numbered in the selection and exposure categories, one study can be rated as up to one star, and comparability can be assigned up to two stars. Higher scores indicate an increase in the quality of the research method. Studies with scores equal to or higher than 6 are considered high-quality studies.

### Data analysis

The heterogeneity in the study was tested by the *Q* test and *I*^2^ [22, 23], and then the random ratio or fixed effect was utilized to merge the odds ratios (OR) and 95% confidence interval (CI) [24]. The significance of the pooled OR was analyzed by *Z* test (*P* < 0.05 judged statistically significant). To estimate the impact of individual studies on aggregate estimates, we also performed sensitivity analysis [25]. Using funnel plot and Eegger’s regression test investigated the publication bias [26, 27]. All data statistical analyses were performed using Stata 12.0 (Stata Corp, College Station, TX, USA).

## Results

### Literature search and study characteristics

**Fig. 1:**
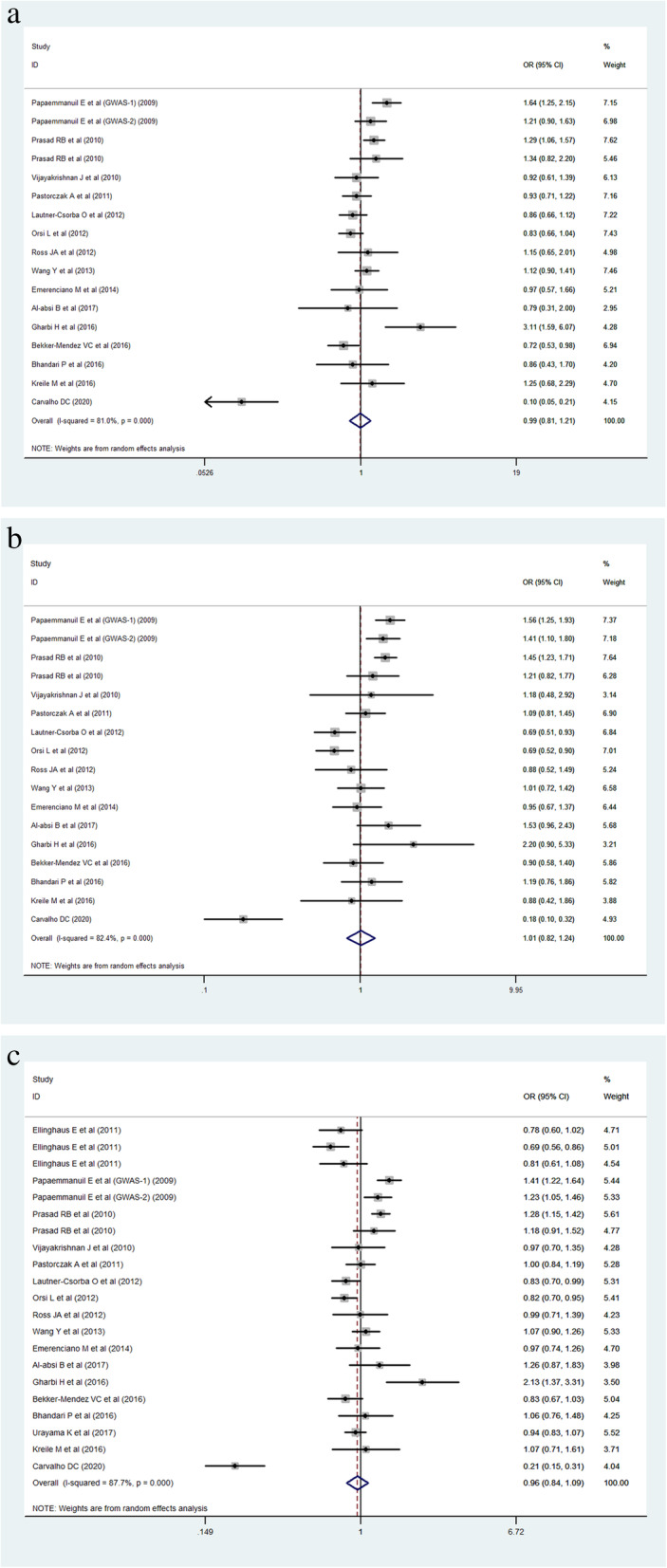
The flow sheet of identification of eligible studies

The flow chart of the literature search was shown in Fig. 1. One hundred sixty-five potentially relevant articles were selected in the preliminary online search. After verifying and deleting 80 duplicate articles, 85 articles entered the final review. Through the review of the title and abstract, 26 articles were included for full-text review. Finally, 16 articles were included in the final study. These studies were published between 2009 and 2017, and 20 studies included 7014 ALL patients and 16,428 controls. The distribution of genotypes in controls in all studies followed HWE. In addition, the NOS scores for all studies ranged from 6 to 8 points, so that the selected articles were considered to be good in methodological quality. The relevant feature information of the included articles was in Tables 1 and 2.

**Table 1:** Characteristic of studies included in the meta-analysis

Author	Year	Country	Ethnicity	Genotype methods	Source of control	NOS score	HWE
Ellinghaus et al.	2011	Germany	Caucasian	SNPlex and TaqMan	HB	7	Does not know
Ellinghaus et al.	2011	Germany	Caucasian	SNPlex and TaqMan	HB	7	Does not know
Ellinghaus et al.	2011	Italy	Caucasian	SNPlex and TaqMan	HB	7	Does not know
Papaemmanuil et al. (GWAS-1)	2009	UK	Caucasian	Illumina arrays	PB	8	0.778
Papaemmanuil et al. (GWAS-2)	2009	UK	Caucasian	Illumina arrays	HB	7	0.517
Prasad et al.	2010	Germany	Caucasian	Kaspar allele-specific PCR	PB	8	0.233
Prasad et al.	2010	UK	Caucasian	Kaspar allele-specific PCR	HB	7	0.310
Vijayakrishnan et al.	2010	Thailand	Asian	Kaspar allele-specific PCR	PB	8	0.162
Pastorczak et al.	2011	Poland	Caucasian	PCR	HB	7	0.454
Lautner-Csorba et al.	2012	Hungary	Caucasian	Sequenom iPlex Gold MassARRAY technology	HB	7	0.508
Orsi et al.	2012	France	Caucasian	Principal component analyses (PCA)	PB	8	0.472
Ross et al.	2012	USA	Caucasian	Taqman	PB	6	0.091
Wang et al.	2013	China	Asian	PCR	HB	7	0.147
Emerenciano et al.	2014	Brazil	Mixed	Taqman	HB	7	0.135
Al-absi et al.	2017	Yemen	Asian	Fluidigm 192.24 Dynamic Array	PB	6	0.149
Gharbi et al.	2016	Tunisia	Caucasian	PCR	PB	7	0.700
Bekker-Mendez et al.	2016	Mexico	Mexican	Taqman	HB	6	0.081
Bhandari et al.	2016	India	Asian	Taqman Illumina	PB	7	0.085
Urayama et al.	2017	Japan	Asian	HumanCoreExome BeadChip	HB	6	Does not know
Kreile et al.	2016	Latvia	Caucasian	PCR-RFLP	PB	6	0.234

**Table 2:** The genotype distribution of *CEBPE* rs2239633

Author	Sample size (case/control)	Female (%) (case/control)	Case					Control				
			CC	CT	TT	C	T	CC	CT	TT	C	T
Ellinghaus et al.	419/474	45.8/–	–	–	–	243	176	–	–	–	246	228
Ellinghaus et al.	406/1682	45.3/–	–	–	–	240	166	–	–	–	841	841
Ellinghaus et al.	287/579	49.5/–	–	–	–	178	109	–	–	–	330	249
Papaemmanuil et al. (GWAS-1)	503/1435	–/–	78	244	181	400	606	332	722	381	1386	1484
Papaemmanuil et al. (GWAS-2)	404/960	–/–	74	188	142	336	472	205	488	267	898	1022
Prasad et al.	1193/1510	44.4/49.9	197	559	437	953	1433	307	773	430	1387	1633
Prasad et al.	183/352	49.2/69.3	26	95	62	147	219	64	183	105	311	393
Vijayakrishnan et al.	190/182	42.6/54.9	103	76	11	282	98	95	78	9	268	96
Pastorczak et al.	388/711	41.2/56.1	119	176	93	414	362	207	344	160	758	664
Lautner-Csorba et al.	543/529	56.2/42.3	173	278	92	624	462	152	256	121	560	498
Orsi et al.	441/1542	46.9/61.0	141	225	75	507	375	432	755	355	1619	1465
Ross et al.	85/363	–/–	19	43	23	81	89	90	165	108	345	381
Wang et al.	568/672	38.6/34.4	245	253	70	743	393	309	281	82	899	445
Emerenciano et al.	160/505	–/48.1	21	68	71	110	210	62	201	220	325	641
Al-absi et al.	136/153	63.2/53.6	10	46	80	66	206	9	70	74	88	218
Gharbi et al.	58/150	44.8/–	15	33	10	63	53	78	59	13	215	85
Bekker-Mendez et al.	285/476	–/52.7	122	128	35	372	198	167	245	64	579	373
Bhandari et al.	162/150	32.7/40.7	21	65	76	107	217	17	69	64	103	197
Urayama et al.	527/3882	–/–				578	476				4138	3626
Kreile et al.	76/121	46.1/–	25	38	13	88	64	46	52	23	144	98

### Meta-analysis results

**Fig. 2:**
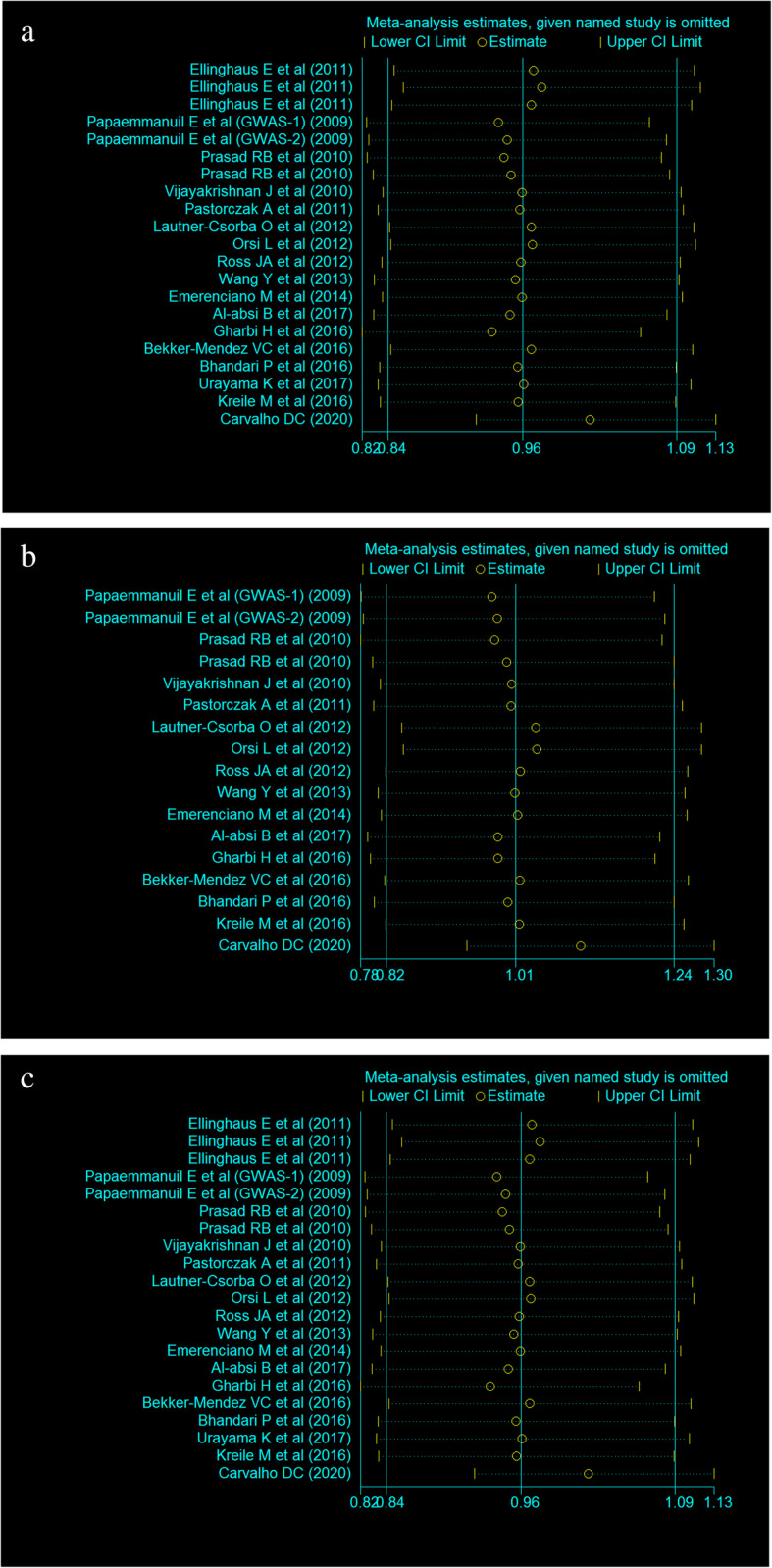
Forest plots of the *CEBPE* rs2239633 polymorphism under different genetic models. **a** The model of CC vs CT + TT. **b** The model of CC + CT vs TT. **c** The model of C vs T

**Fig. 3:**
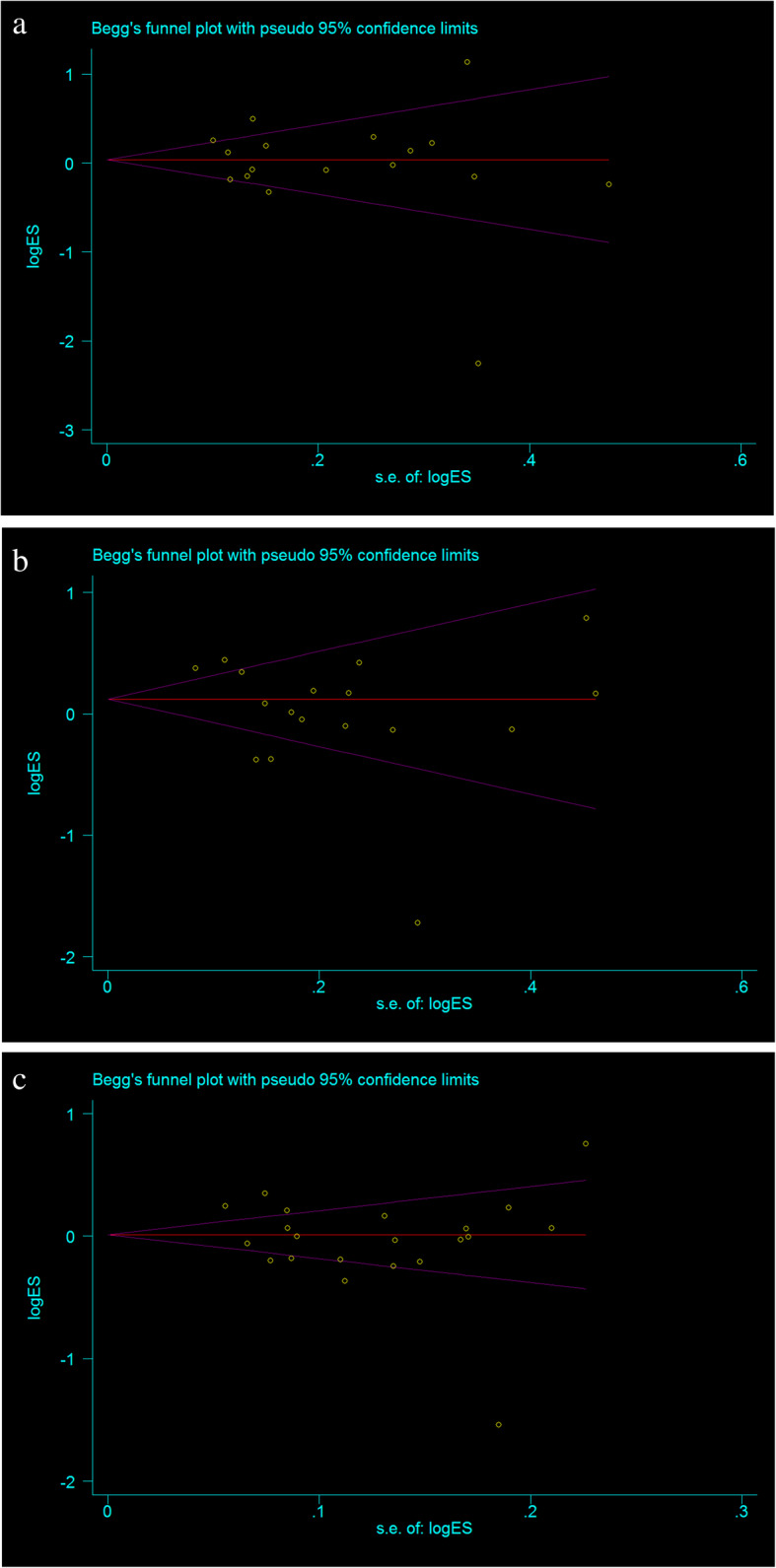
Sensitivity analysis examining the association between the *CEBPE* rs2239633 polymorphism and risk of childhood ALL under these models (CC vs CT + TT, CC + CT vs TT, C vs T)

**Fig. 4:**
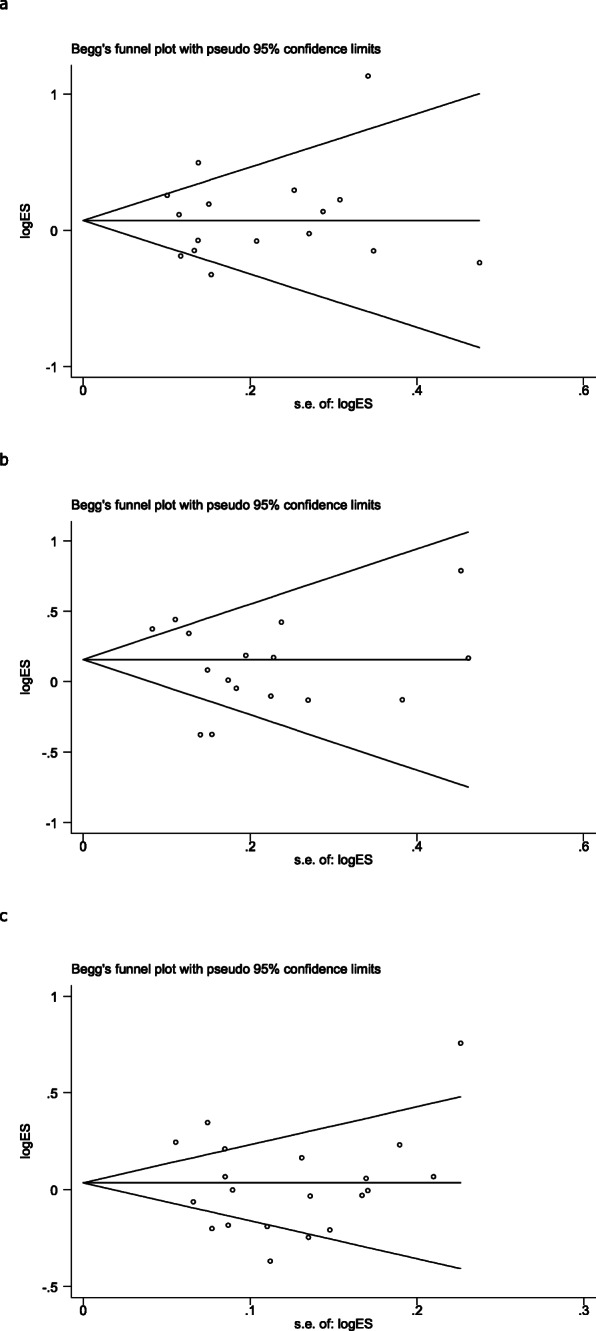
Begg’s funnel plot for publication bias analysis. **a** The model of CC vs CT + TT. **b** The model of CC + CT vs TT. **c** The model of C vs T

The heterogeneity of the three genetic models was determined by *Q* test and *I*^2^ statistics. As shown in Fig. 2, these were serious heterogeneity in the three models (CC vs CT + TT: *P* < 0.001, *I*^*2*^ = 63.6%; CC + CT vs TT: *P* = 0.002, *I*^*2*^ = 70.2%; C vs T: *P* < 0.001, *I*^*2*^ = 79.2%); thus, we used the random-effect model to analyze the three models. Our results did not find significant associations between *CEBPE* rs2239633 polymorphism and the risk of ALL under the three models, CC vs CT + TT (OR = 1.08, 95% CI = 0.94–1.26, *P* = 0.280), CC + CT vs TT (OR = 1.10, 95% CI = 0.94–1.30, *P* = 0.228), and C vs T (OR = 1.02, 95% CI = 0.92–1.13, *P* = 0.752). In the subgroup analysis by ethnicity, no significant association was found in three models in both Caucasian and Asian populations (Table 3). Sensitivity analysis was used to assess the impact of each individual study on the pooled OR by sequentially removing each eligible study. Our results suggest that none of the studies affected the overall outcome of the pooled OR (Fig. 3). Begg’s funnel plot was used to assess publication bias, and the results showed that publication bias was not reflected in the three genetic models (CC vs CT + TT: *P* = 0.742; CC + CT vs TT: *P* = 0.285; C vs T: *P* = 0.560) (Fig. 4).

**Table 3:** Summary of pooled OR in different ethnicities

Genetic model	Group	Pooled OR (95% CI)	Heterogeneity		Test for overall effect	
			*P*	*I*^2^	*Z*	*P*
CC vs CT + TT	Caucasians	1. 17 (0.97–1.41)	< 0.01	68.9%	1.68	0.092
	Asia	1.04 (0.87–1.25)	0.701	0.0%	0.43	0.666
CC + CT vs TT	Caucasians	1.09 (0.89–1.35)	< 0.01	78.7%	0.85	0.393
	Asia	1.17 (0.94–1.47)	0.583	0.0%	1.38	0.168
C vs T	Caucasians	1.03 (0.89–1.18)	< 0.01	84.3%	0.36	0.718
	Asia	1.00 (0.92–1.10)	0.538	0.0%	0.10	0.917

## Discussion

As a transcription factor specifically expressed in myeloid cells, CCAAT/enhancer-binding protein-ε (CEBPE) played an important role in the proliferation, growth, differentiation, and apoptosis of myeloid cells and participates in the transcriptional regulation of a series of myeloid-specific genes. Loss of activity was an important factor leading to the onset of bone marrow disease [28]. In recent years, a growing number of published studies had investigated the relationship between *CEBPE* rs2239633 polymorphism and ALL risk [29–37]. It also included some meta-analysis, but the results obtained from the meta-analysis were contradictory and conflicting. To further assess the relationship between *CEBPE* rs2239633 polymorphism and ALL risk, we performed an updated meta-analysis to investigate the relationship between *CEBPE* rs2239633 polymorphism and ALL risk.

Although the GWAS study by Papaemmanuil et al. [6] proved that the 5′ SNP rs2239633 located in *CEBPE* has a strong correlation with children’s ALL in the European population, however, this meta-analysis showed that no significant association was found in the three selected genetic models. In the subgroup analysis of ethnicity, no significant correlation was found under the three genetic models. On the one hand, this difference may be caused by the linkage imbalance between populations. There are also some differences between the population samples. On the other hand, the exact pathogenesis of *CEBPE* in the etiology of leukemia was still unclear. The CEBPE mutation may have different effects on the immune system of different children.

Previously, a meta-analysis was performed for 11 case-control studies with 5639 cases and 10,036 controls by Wang et al. [13], the results showed no association of the *CEBPE* rs2239633 polymorphism and childhood ALL risk, and subgroup analysis stratified by ethnicity found a significant association of this polymorphism with childhood ALL in the Caucasian subgroup and Hispanic subgroup, but not in the Asian subgroup. Sun et al. [14] also conducted a meta-analysis, including 22 published studies involving 6152 patients and 11,739 healthy controls, and the results also showed that *CEBPE* rs2239633 variant was associated with decreased risk of childhood B cell ALL in Europeans, but not among T cell ALL, Asian, and mixed populations. The results of the two meta-analyses are diametrically opposed, and this difference may be due to the difference in the number of samples included and the sample size. This study combines the latest research literature with the first two meta-analyses to more fully describe the relationship between *CEBPE* rs2239633 and CALL. In terms of statistical power, it is significantly better than the previous meta-analysis of Sun et al. [14] and Wang et al. [13].

However, there are certain limitations in our research. First, databases that include only published research in both Chinese and English are selected for analysis, and other languages or unpublished potential researches may be missed. Second, due to the lack of raw data, we were unable to assess potential interactions of gene-genes and genes-environments. Third, the meta-analysis includes data from Europeans and Asians, so the results of this item apply only to these two ethnic groups. Fourth, among the three models, heterogeneity may greatly influence the conclusion of the meta-analysis. Lastly, maybe the results obtained from this study have limited clinic significance, but we think that the current study was needed and meaningful for understanding the relationship between the CEBPE rs2239633 polymorphism and the CALL susceptibility.

## Conclusion

In summary, our study showed that the *CEBPE* rs2239633 gene polymorphism did not increase or decrease the risk of susceptibility to CALL. Although the specific causes of childhood leukemia were still unclear, a large number of existing researches tended to suggest that the occurrence of childhood ALL was the result of a combination of factors, especially the genetic and environmental factors. Therefore, in the future, when studying the relationship between *CEBPE* rs2239633 polymorphism and childhood ALL, the influence of environmental factors on the relationship between the two should be removed.

## Data Availability

The datasets generated and/or analyzed during the current study are available from the corresponding author on reasonable request.
